# Parallel charge sheets of electron liquid and gas in La_0.5_Sr_0.5_TiO_3_/SrTiO_3_ heterostructures

**DOI:** 10.1038/srep18282

**Published:** 2015-12-16

**Authors:** X. Renshaw Wang, L. Sun, Z. Huang, W. M. Lü, M. Motapothula, A. Annadi, Z. Q. Liu, S. W. Zeng, T. Venkatesan, Ariando

**Affiliations:** 1 NUSNNI-Nanocore, National University of Singapore, 117411 Singapore; 2 Department of Physics, National University of Singapore, 117542 Singapore; 3 Faculty of Science and Technology and MESA+ Institute for Nanotechnology, University of Twente, P.O. BOX 217, 7500 AE Enschede, The Netherlands; 4 Department of Electrical and Computer Engineering, National University of Singapore, 117576 Singapore; 5 National University of Singapore Graduate School for Integrative Sciences and Engineering (NGS), 28 Medical Drive, Singapore 117456, Singapore

## Abstract

We show here a new phenomenon in La_0.5_Sr_0.5_TiO_3_/SrTiO_3_ (LSTO/STO) heterostructures; that is a coexistence of three-dimensional electron liquid (3DEL) and 2D electron gas (2DEG), separated by an intervening insulating LSTO layer. The two types of carriers were revealed through multi-channel analysis of the evolution of nonlinear Hall effect as a function of film thickness, temperature and back gate voltage. We demonstrate that the 3D electron originates from La doping in LSTO film and the 2D electron at the surface of STO is due to the polar field in the intervening insulating layer. As the film thickness is reduced below a critical thickness of 6 unit cells (uc), an abrupt metal-to-insulator transition (MIT) occurs without an intermediate semiconducting state. The properties of the LSTO layer grown on different substrates suggest that the insulating phase of the intervening layer is a result of interface strain induced by the lattice mismatch between the film and substrate. Further, by fitting the magnetoresistance (MR) curves, the 6 unit cell thick LSTO is shown to exhibit spin-orbital coupling. These observations point to new functionalities, in addition to magnetism and superconductivity in STO-based systems, which could be exploited in a multifunctional context.

The surface or interfacial conductivity in transition metal oxides is a potential candidate for and has been exploited in multifunctional oxide-based electronics^1^,^2^. Strontium titanate (SrTiO_3_ or STO) occupies a central position in this voyage, both on account of its intrinsic properties and its suitability as a substrate for all-oxide heterostructures, or as a buffer layer for oxide heterostructures built on silicon. In STO, the conducting channels are generally created by introducing cationic dopants^3^,^4^, inducing oxygen vacancies^5^ or depositing a polar overlayer such as LaAlO_3_ (LAO)^6^, LaTiO_3_^7^, LaVO_3_^8^, ZrO_2_:Y_2_O_3_^9^ or Al_2_O_3_^10^. Particularly, the 2DEG between insulating polar LAO and non-polar STO^6^ has attracted the most attention, due to the emergence of unexpected properties, including superconductivity^11^, magnetism^12^,^13^, electronic phase separation^14^,^15^, MIT^15^ strain response^16^, Lifshitz transition^17^, spin-orbital coupling^18^ and multi-types of carriers^19^,^20^. Since the first demonstration of the 2DEG at the LAO/STO interface, extensive research effort has been mostly focused on exploration of the underlying mechanism of the 2DEG, with a special attention on the role of the Ti atoms, which can co-exists in two different valence states of Ti^3+^ and Ti^4+^^21^,^22^. At the interface, the formation of Ti^3+^ is expected due to the electronic reconstruction needed to avert diverging potential introduced by the polar layer, and the Ti orbitals will split into a large number of Ti sub-bands^22^, whose degeneracy and electronic occupancy are sensitive to the details of the disorder^5^ and biaxial strain of the interface^16^.

In this report, we focus on the properties of atomically flat La doped STO films grown on different substrates. La doped STO has a wide range of intriguing applications, such as transparent conductors^23^, conductive buffer layer for high temperature superconductor^24^, anodes for solid oxide fuel cells^25^, harboring high mobility electrons in confined quantum well^5^, as well as sensors^26^. La doped STO is a single-band conductor^27^ with expected conductivity due to La doping. By doping La into STO, La doped STO shows filling dependent Fermi liquid behaviour^27^. By varying the ratio of La to Sr, Sr_1-X_La_X_TiO_3_ changes from a Mott insulator (x = 1, LaTiO_3_) to a single-band conductor, and further to a band insulator (x = 0, STO). Structurally, La dopant transforms Sr_1-X_La_X_TiO_3_ from Pm3m (x = 0) to Ibmm (0.2 ≤ x < 0.7) and to Pbnm (0.7 < x ≤ 1)^28^. The case for 50% La doped STO is particularly interesting, due to the Ti valences. When doping 50% of La into STO, dopants in a stoichiometric LSTO render Ti into two valence states^27^ (50% of Ti^3+^ and 50% of Ti^4+^). This turns LSTO into a polar compound with an alternating polarity of +0.5e and −0.5e (La^3+^_0.5_Sr^2+^_0.5_O^2-^ and Ti^3+^_0.5_Ti^4+^_0.5_O^2−^_2_)^29^. Following the prediction of the polar catastrophe model^21^, the alternating polarity should lead to a charge transfer from LSTO to STO surface (assuming there is no screening by the free electrons), and the electrons at STO surface would exhibit properties similar to those at the LAO/STO interface. However, LSTO has to be an insulator to satisfy the requirements of the polar catastrophe. By applying biaxial strain, we were able to transform the electrical properties of thin LSTO to an insulating state, and to generate two conducting channels in the system; one is in the LSTO film and the other at the interface between LSTO and STO. Various LSTO films were prepared on different substrates and behaviour of the two channels of carriers for different LSTO thicknesses at different temperatures, and under the influence of applied magnetic and electric fields were studied.

## Results

### Temperature dependence of transport property

**Figure 1:**
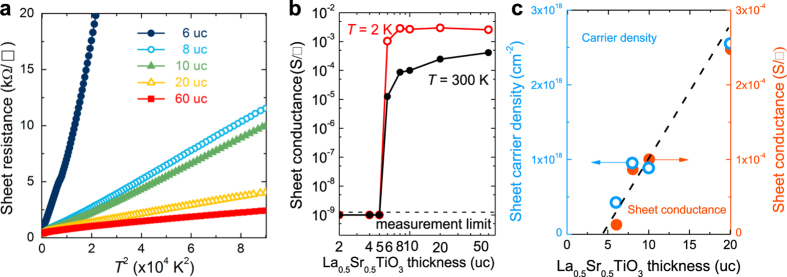
Abrupt MIT. (**a**) Quadratic function between sheet resistance and temperature indicates an electron liquid behaviour. (**b**) Sheet conductance versus thickness measured at 2 and 300 K showing a more than 6 orders of magnitude change between 5 and 6 uc samples. (**c**) Zoom-in of sheet conductance and sheet carrier density as a function of thickness measured at room temperature. The extrapolated dash line is guide to the eyes and that suggests an insulating layer of 5 uc.

The sheet resistance *R*_s_ of all LSTO films were measured in a Van der Pauw geometry. In these measurements, Aluminum contacts are created by ultrasonically bonding aluminum wires that penetrate through the LSTO film down to the interface between LSTO and STO, allowing the electrical probing of both the film and interface using the same wire. Figure 1a summarizes their temperature dependence, which shows a *T*^2^ dependence. This is typical of EL system where electron-electron scattering dominates. Figure 1b shows the sheet conductance as a function of film thickness at 2 and 300 K, suggesting a sharp MIT with a critical thickness of 6 uc. When the thickness is less than 6 uc, LSTO films turn into an insulating phase with a conductance below our measurement limit. While the sheet conductance progressively increases as film thickness increases at 300 K, it jumps by more than six orders of magnitude when the thickness reaches 6 uc at 2 K. The sheet conductance is almost constant at low temperature (2 K) and this might be a result of the dominating scattering induced by structural defects in LSTO due to the STO structural phase transition at 105 K. Figure 1c shows the sheet conductance and carrier density of LSTO films for different thicknesses at 300 K obtained by Hall measurements (discussed in the next section). Within the first 20 uc, all data points fall onto a line intersecting zero conductance at 5 uc. In other words, the data extrapolates to an insulating layer of 5 uc, which is consistent with the critical thickness of 5 uc (Fig. 1b). The abrupt MIT in LSTO/STO is particularly interesting due to the more than 6 orders of magnitude change in conductance within one uc in contrast to the more gradual MITs observed in other conducting oxides, such as LaNiO_3_^30^ and SrVO_3_^31^.

### Nonlinear Hall resistance

Further investigations were conducted by performing Hall measurement. While the 6 uc LSTO/STO sample shows a linear Hall resistance (*R*_xy_), samples with a thickness greater than 6 uc show a nonlinear *R*_xy_ at low temperatures (<100 K). There are four possible origins for nonlinear *R*_xy_: (i) quantum effect due to strong magnetic field above the weak field limit, (ii) anomalous Hall effect due to ferromagnetism, (iii) multi-band nature of materials which leads to different types of carriers in the material, and (iv) spatially separated multi-channel of carriers with different mobilities. The weak field limit, above which quantum effect can be observed, can be expressed as:


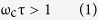


where ω_c_ is cyclotron frequency and τ is the mean time between scattering events. Considering ω_c_ = *e*B/m*, where B is the applied magnetic field and m* is the effective mass, and electron mobility *μ* = *e*τ/m*, the weak field limit can be rewritten as:


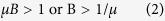


**Figure 2:**
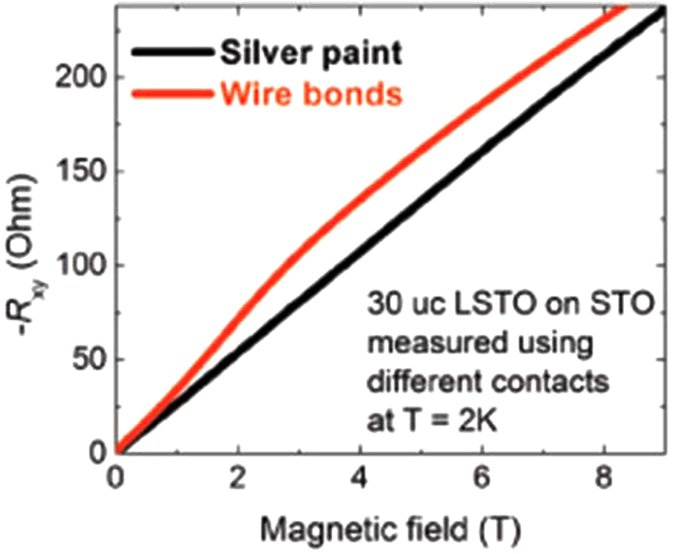
Comparison of Hall resistance measured in two different types of contacts. Hall resistance *R*_xy_ of a 30 uc LSTO/STO is measured at 2 K using two different contact methods. Silver contacts, which electrically contact only the LSTO, show linear *R*_xy_. Wire bonds, which contact both LSTO and the interface of LSTO/STO, show nonlinear Rxy.

This sets a limit for the applied magnetic field above which the Hall resistance can become nonlinear. Considering the linear low field (below 2.5 T) of *R*_xy_ gives Hall mobility values for our samples ranging from 200 cm^2^V^−1^s^−1^ (300 K) to 900 cm^2^V^−1^s^−1^ (2 K), corresponding to a lowest possible weak field limit of 1/*μ* = 1/(900 cm^2^V^−1^s^−1^) = 11.1 T. This is much higher than the maximum applied magnetic field in our measurements, thus the possibility of quantum effect in the system is ruled out. In order to examine the possibility of anomalous Hall effect, we performed bulk M(*H*) magnetization measurement using SQUID, MR measurement at 2 K, and scanning SQUID microscope imaging at 4 K (see Supplementary Information). There is no indication of ferromagnetism in all three measurements. We thus eliminate the possibility of anomalous Hall effect. The multi-band nature is also unlikely to be responsible for the nonlinear *R*_xy_ in our heterostructures for four reasons. Firstly, LSTO has been shown to be a single-band material^27^. Secondly, we were able to tune the 60 and 300 uc LSTO samples by applying electric field using STO substrate as the dielectric back gate (see Supplementary Information). This is in line with multi-channel model in which low carrier density channel at interface can be tuned by back gate and high carrier density channel cannot. Thirdly, we also compared Hall resistance measured by two different types of electrical contacts (Fig. 2). The wire bond contacts are created by ultrasonically melting Al wire. The energy created during melting Al enables Al wire to penetrate through LSTO film and electrically contacting the interface region of LSTO/STO heterostructures. Silver paint contacts are created by gently painting silver paint onto the surface of LSTO/STO heterostructures, and are only able to electrically contact the LSTO thin film. As shown in Fig. 2, *R*_xy_ measured using silver paint contacts is linear and the one measured using ultrasonic wire bonds is nonlinear. The contact comparison experiment confirmed that LSTO is a single band conductor, and it is also in line with our proposed two channel model. The *R*_xy_ measurements using this configuration show a linear magnetic field dependence, consistent with a single band nature of LSTO. Fourthly, a 50 uc LSTO grown on LAO shows linear *R*_xy_ (see Supplementary Information). Therefore, we conclude that the origin of the nonlinear *R*_xy_ is the presence of multi-channel carriers^32^ which are spatially separated.

**Figure 3:**
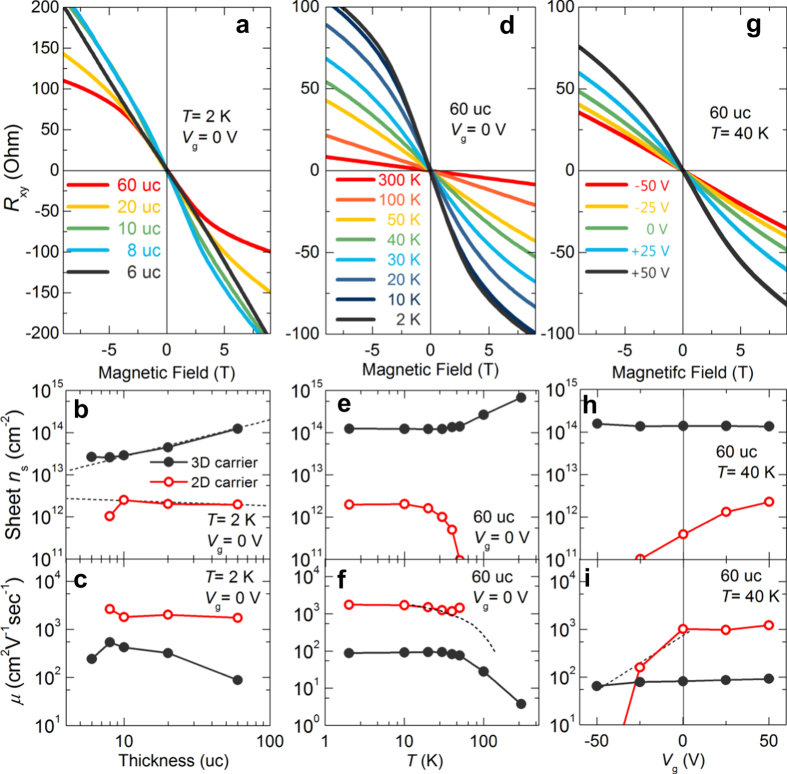
Nonlinear Hall effect in LSTO films. (**a–c**) Evolution of Hall resistance *R*_xy_, carrier density and mobility as functions of LSTO thicknesses. All data are collected at 2 K with no back gate voltage (*V*_g_) applied. The *R*_xy_ is linear for 6 uc, but becomes increasingly nonlinear as the film thickness grows. (**d–f**) Temperature dependence of nonlinear Hall effect, carrier density and mobility for 60 uc LSTO. Nonlinear Hall effect was only observed under 100 K. (**g**–**i**) *V*_g_ dependence of nonlinear Hall effect, carrier density and mobility for 60 uc LSTO measured at 40 K.

To further confirm the existence of multi-channel carriers, we performed a multi-channel fitting on the transport data for different thickness, temperature and gate voltage. Figure 3a shows a set of *R*_xy_ measured at 2 K for different film thicknesses. The nonlinearity in *R*_xy_ starts to appear for an 8 uc sample and becomes more evident for thicker films, while a linear *R*_xy_ is observed in 6 uc samples. Hence, the 6 uc LSTO/STO has only one type of carriers. The properties of the two types of carriers in samples thicker than 6 uc can be extracted through curve-fitting of the nonlinear *R*_xy_ with a multi-channel model^32^. For a two-channel case, *R*_xy_ can be described as


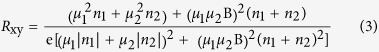


where *n*_1_, *μ*_1_, and *n*_2_, *μ*_2_ are the density and mobility of the two types of carriers, respectively.

As shown in Fig. 3b,c, the density of one of the carriers (*n*_1_, indicated by black dots) is an order of magnitude higher than that of the other (*n*_2_), while its mobility (*μ*_1_) is significantly lower than its counterpart (*μ*_2_). The *n*_*1*_ starts to appear in the fitting results when LSTO is 6 uc, and it increases proportionally with film thickness while *μ*_*1*_ decreases. This indicates that *n*_*1*_ originates from La doping in the LSTO film^27^. (Because the La dopants are in the LSTO film, we use “3D” to denote *n*_1_ carrier in analogy to the bulk doping effect.) On the other hand, low density *n*_2_ starts to appear at 8 uc. *n*_2_ and *μ*_2_ also show little dependence on film thickness, which is a manifestation of its 2D nature. However, the “3D” carrier density for 30 uc sample at room temperature is about 8 × 10^14^ cm^−2^. Considering effective thickness of 25 uc and 0.39 nm as *c* axis lattice parameter, this measured carrier density is only 10.3% of expect carrier density from 50% Sr doping. The unexpected low carrier density is possibly due to different reasons which require further investigation, namely (i) surface depletion which can be as thick as 16.5 nm and 5.3 nm at 2 K and 200 K respectively in 5% La doped SrTiO_3_ films grown on SrTiO_3_ substrate^33^; (ii) increase on the thickness of interval insulating layer as increasing total film thickness; (iii) depletion originated from SrTiO_3_ substrates and one example on LaTiO_3_/SrTiO_3_ heterostructure is discussed in ref. 34; and (iv) structurally ordered phases of La_2_Sr_n−2_TiO_3n+1_^35^.

In order to investigate the scattering processes, temperature dependence of *R*_xy_ was studied with film thickness fixed at 60 uc (Fig. 3d). Nonlinear *R*_xy_ was only observed when temperature drops below ~100 K. The temperature dependence of the *n*_*1*_and *μ*_1_ suggests that the 3D carriers exhibit an EL nature. In an EL system, electron-electron interaction dominates, thus the electron mobility inherently increases with decreasing density, as is the case for *n*_1_ and *μ*_1_ plotted in Fig. 3e,f. The 2D carriers vanishes when increasing temperature above ~100 K. Furthermore, while *n*_2_ varies by more than an order of magnitude over the range of 100 to 10 K, *μ*_2_ shows little change, which points towards electron gas behaviour. The temperature dependence of both carriers in our system is similar to that observed in other systems^8^,^19^,^36^,^37^. The increase in carrier concentration of 2DEG at low temperature has also been observed in Nb-doped STO^38^, and is yet to be understood. The decrease in carrier mobility of the 3DEL at high temperatures is due to phonon scattering^38^,^39^.

The temperature dependence of Hall resistance shows that the low carrier density carrier starts to emerge at ~40 K (see Fig. 3e,f). Thus, electric field effect experiments were also performed at 40 K in order to see whether the carriers can be turned on and off by the field. Even though it is still lower than that at 2 K, STO at 40 K still has a relatively large dielectric constant of ~4000^39^,^40^,^41^, which is sufficient in inducing significant changes in nonlinear Hall resistance. The leakage current from drain to gate was well below 100 nA range and the source-drain current was fixed below 1 *μ*A. The back gate experiment also answers an important question: is the 2DEG located at the top surface of the LSTO film or at the interface of LSTO/STO heterostructures? With the 0.5 mm STO substrate as the dielectric material, the 2DEG shall be tunable by the electrical field only if it were to be located at the interface between the conducting LSTO film and STO substrate. Additionally, the carrier density *n*_2_ should increase when the back gate voltage increases. If the 2D carriers are at the top surface of LSTO film, the back gate voltage will not be able to tune *n*_2_ due to the screening effect by the thick metallic LSTO film. Thus we conclude that the 2DEG is at the surface of STO substrate. As the back gate voltage (V_g_) increases from −50 to 50 V, *n*_2_ and *μ*_2_ increase, while *n*_1_ and *μ*_1_ remain constant. These field effect dependencies of the two types of carriers and the fact that LSTO films have a 6 uc critical thickness (*i.e*. the 5 uc or lower LSTO films are insulating) point to the conclusion that the two types of carriers are spatially separated by an insulating intervening layer in the LSTO/STO heterostructure. There are two charge sheets in LSTO/STO heterostructures, namely a high density but low mobility 3DEL in the LSTO film and a high mobility but low density 2DEG at the interface between LSTO film and STO substrate.

### Effect of Strain

**Figure 4:**
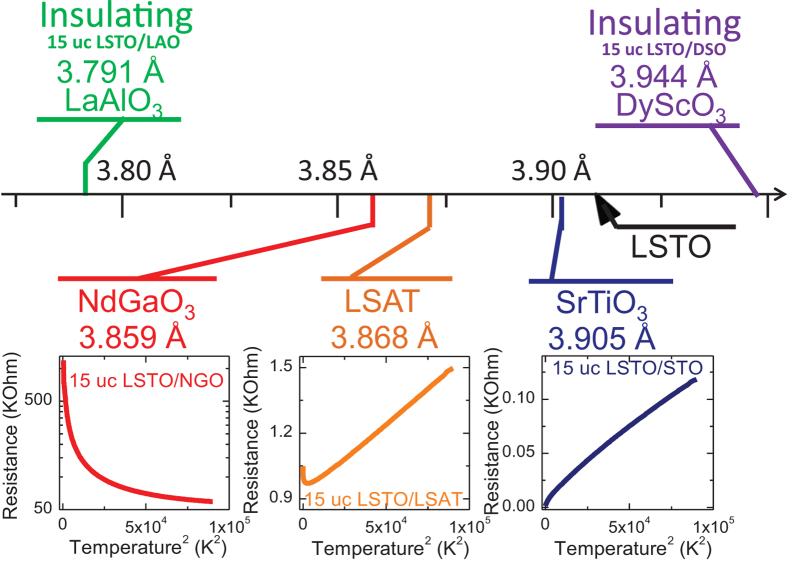
Effect of strain on resistance of 15 uc LSTO grown on different substrates. Resistance versus temperature of 15 uc LSTO grown on different substrates. With increasing compressive strain, the conductivity evolves gradually from conducting to localization at low temperature, to semiconducting, and eventually to insulating. With tensile strain, the system loses conductivity easily.

In order to understand the origin of the insulating intervening LSTO layer, 15 uc LSTO films were grown on five different substrates, namely LAO (rhombohedral, 3.791 Å), NdGaO_3_ (110) (NGO, orthorhombic, 3.859 Å), (LaAlO_3_)_0.3_(Sr_2_AlTaO_6_)_0.7_ (LSAT, cubic, 3.868 Å), STO (cubic, 3.905 Å) and DyScO_3_ (110) (DSO, orthorhombic, 3.944 Å)^42^,^43^. All films are of good quality and have surface peak-to-peak roughness of 1 uc. Figure 4 summarizes the temperature dependence of the sample resistance. Among those substrates, the least compressive strain (0.13%) is provided by STO, and this results in a metallic LSTO with the lowest resistance. As the amplitude of compressive strain increases (from STO, LSAT, NGO to LAO), the metallic state is gradually suppressed. While the compressive strain introduced by LSAT (1.07%) only leads to weak localization behaviour at low temperatures, slightly larger compressive strain from NGO (1.30%) causes a complete semiconducting behaviour. The highest 3.04% compressive strain in the LSTO/LAO system turns the film into a complete insulator. On the other hand, the tensile strain introduced by DSO, despite its relatively small value (0.87%), turns the LSTO film to an insulator. From the extreme sensitivity of the electron transport behaviour of ultrathin LSTO films against strain, we conclude that the strain is one of the possible origins for the 5 uc insulating layer in the LSTO thin films deposited on STO. Typically, oxide thin films takes tens of unit cells to relax the (~3%) strain effect from the substrate. In the LSTO/STO case, considering the small amount of compressive strain (0.13%), 5 uc layer is a reasonable number to expect for LSTO to relax to its bulk lattice parameters.

### Weak anti-localization

**Figure 5:**
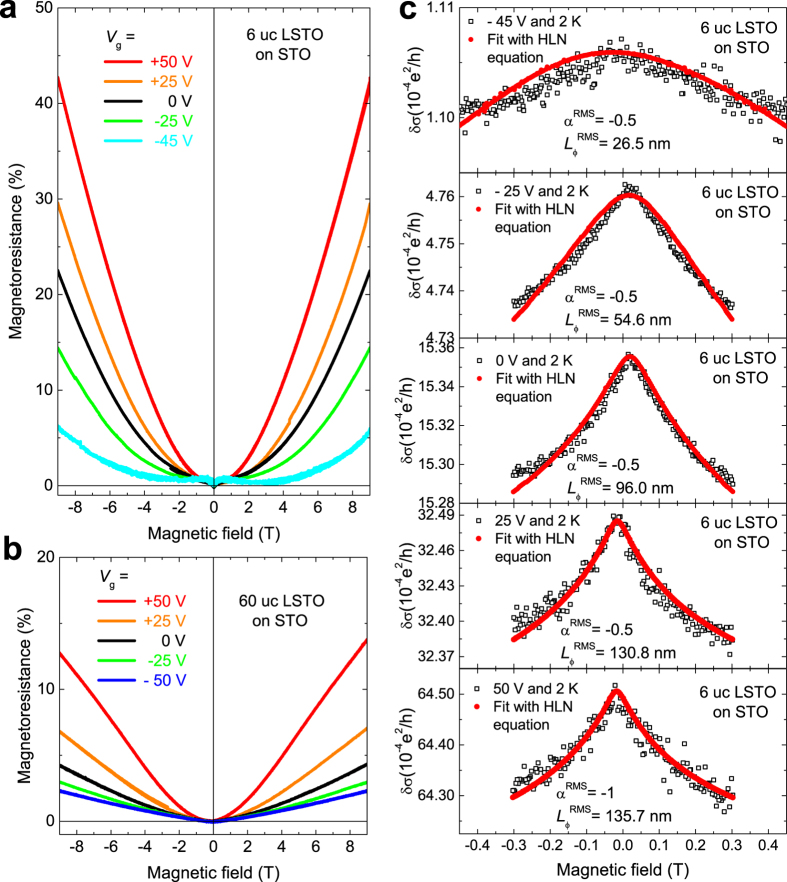
Magneto-transport studies of 6 uc LSTO/STO heterostructure. Magnetoresistance of (**a**) 6 uc LSTO/STO heterostructures and (**b**) 60 uc LSTO/STO heterostructures under different back gate voltages. Weak anti-localization is observed in 6 uc LSTO/STO heterostructures, indicating the existence of spin-orbital coupling. (**c**) Hikami–Larkin–Nagaoka (HLN) fitting on the spin-orbital coupling in 6 uc LSTO/STO heterostructures under different gate voltages.

The magnetic field dependence of the carriers was investigated by MR measurements on both 6 uc (Fig. 5a) and 60 uc (Fig. 5b) samples under different back gate voltages (*V*_g_). In large magnetic fields, all measured LSTO samples show positive MR. However, as shown in Fig. 5a, the 6 uc LSTO/STO shows WAL under all *V*_g_, while the 60 uc LSTO/STO sample does not show any sign of WAL. Since 6 uc LSTO/STO heterostructure has only a thin layer of La-induced EL on the surface of LSTO, the WAL in 6 uc LSTO provides information on dimension confinement effect on the electron liquid. For a 2D system in a perpendicular magnetic field, WAL can be fitted by the Hikami-Larkin-Nagaoka (HLN) formula^44^,^45^:


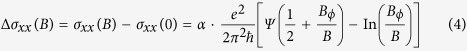


where 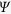 is the digamma function, 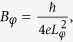 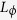 is the dephasing length, and α is governed by the interaction symmetries. Using metal-oxide-semiconductor field-effect transistor (MOSFET) as a prototype, Hikami *et al*. discovered that the value of α in Eq. 4 is 1 for orthogonal case, 0 for unitary case and −0.5 for symplectic case^44^. In the symplectic case, the system has strong spin-orbit interaction and no magnetic scattering. Recently, it was also reported that α can be −1 = −(1/2 + 1/2) in a top gated topological insulator Bi_2_Se_3_, reflecting a change in the effective number of coherent channels^46^. In our study, these coherent channels are associated with the surface and interface charge sheets. As shown in Fig. 5c, the MR behaviours of the 6 uc sample are modulated by the electric field effect and are well fitted by the HLN formula, confirming the existence of spin-orbit coupling in the 6 uc LSTO/STO. As the *V*_g_ varies from 25 to −45 V, the HLN fitting returns an α = −0.5, indicating a single coherent channel. This is consistent with the conclusion we drew from our Hall effect results. When the *V*_g_ is set at 50 V, α becomes −1, indicating the appearance of two independent coherent channels. This is due to the formation of an additional channel at the interface under sufficiently large gate voltage, similar to the switching of 2DEG in 60 uc LSTO/STO using back gate experiments presented above. As the film thickness increases, however, the bulk LSTO dominates and the WAL is masked as shown in Fig. 5b for the 60 uc case, where the WAL cusp at low magnetic field is absent. This is highly possible due to the change of dimensionality of the top charge sheet from 2D to 3D^47^,^48^.

## Discussion

### Configuration of the charge sheets

Our results show that the 2DEG is at top surface of STO substrate and the 3DEL is in the LSTO film. The location of the 2DEG is primarily confirmed by electric field effect experiments on LSTO/STO heterostructures with LSTO thicknesses of 5, 60 and 300 uc (see Supplementary Information). The density of 3DEL in LSTO thin film is consistently proportional to the LSTO film thickness. Due to the strain induced by STO, a 5 uc insulating intervening LSTO layer is formed and it spatially separates the two charge sheets. The 5 uc LSTO is found to be insulating in all different thicknesses of the LSTO film on STO.

### Mechanism of multi-channel charge sheets

The two types of carriers and their behaviour are commonly observed in STO-based heterostructures and superlattices, including LaVO_3_/STO heterostructure^8^, LAO/STO heterostructure^19^, LSTO/STO superlattice^33^, LTO/STO superlattice^37^. The low-density and high-mobility carriers are always located at the STO side, primarily due to the strong dielectric screening by high dielectric constant of STO at low temperatures. In all of these systems, the temperature dependence of the carriers shows similar behaviour: high-density carriers associate with thermal activation, while low-density carriers start to be observed only from around 100 K and lower. More strikingly, the low-density high-mobility 2DEG carriers have opposite trends to those at LAO/STO interface. More investigations are still required to understand the electron behaviours in these classes of systems.

In our LSTO/STO heterostructures, polarization in LSTO is most likely the origin for the appearance of 2DEG on the STO side. It has been proposed that “charge leakage” effect can be the origin of conductivity in LTO/STO system^49^. However, “charge leakage” contradicts the thickness dependence MIT observed in LSTO/STO heterostructures. Furthermore, all STO-based multi-carrier heterostructures (LaVO_3_/STO heterostructure^8^, LAO/STO heterostructure^19^, LSTO/STO superlattice^33^ and including charge leakage system of LTO/STO superlattice^37^) are consisting of a polar over layer. This indicates that polarization plays a crucial role in the formation of the 2DEG.

### Spin-orbital coupling

The spin-orbit coupling has been used to create and control spin-related properties in many material systems, such as topological insulators^46^,^50^,^51^, conventional semiconductor heterosctructures^52^,^53^, and LAO/STO oxide interfaces^18^,^54^. According to ref. 55, sub-bands structure in a quantum well is able to induce spin-orbit interaction^55^ and electrons in the 6 uc LSTO/STO occupy different *d* orbitals (sub-bands) of the Ti atoms^56^,^57^. We therefore hypothesize that the spin-orbital coupling at 6 uc LSTO/STO is due to the intrinsic spin-orbit coupling in the doped-STO^55^,^56^. The proposed model agrees with vanishing of spin-orbit coupling in thick 60 uc LSTO and the tunability of spin-orbit coupling in 6 uc LSTO with electric field. Increasing the thickness increases the dimensionality of the quantum well from 2D to 3D and back gated fields influence the occupancy of the sub-bands. The tunable spin-orbit coupling in LSTO/STO heterostructures could be a potential platform for creating new spin-related functionalities.

Furthermore, our two channel model only presents a simplified picture of the transport properties in LSTO/STO heterostructures. The complicated nature of the thin film form of La doped STO might originate from strong correlation involving, but not limited to, multi-band feature^58^, spin-orbital coupling^59^, charge leakage^60^. First principles calculations will be very helpful to further understand the system.

In summary, we observed an abrupt MIT without an intermediate semiconducting state in LSTO grown on STO when the LSTO film thickness is decreased from 6 to 5 uc. From our nonlinear *R*_xy_ study with films thicker than 8 uc, two charge sheets are characterized as a 3DEL in the LSTO film and a 2DEG at the LSTO/STO interface. The transport properties of LSTO films on different substrates demonstrate that a 5 uc insulating layer, which spatially separates the two types of carrier, is created in the LSTO/STO heterostructure by the lattice mismatch between LSTO and STO. Further MR measurement of the 6 uc heterostructure shows WAL. Based on the HLN fitting on the MR, a gating dependent spin-orbit coupling is shown. As the film thickness increases, an extra channel of 2DEG builds up at the interface, carriers in LSTO films evolve into a 3DEL and the spin-orbit coupling is lost. Our results suggest a novel way of creating exotic properties and manipulating electronic property through strain engineering.

## Methods

### Target preparation

A polycrystalline LSTO target used for the thin film depositions were prepared from high purity La_2_O_3_ (99.999%), TiO_2_ (99.999%) and SrO (99.9%) powders. The powders were carefully weighed, mixed and ground for several hours before being sintered at 600 °C for 8 hours and sintered again at 900 °C for 10 hour. Next, the powders were ground into powder, pressed into pellets and calcinated at 1300 °C for 36 hours.

### Substrate preparation

To achieve single-terminated surfaces for STO, the substrates (CrysTec GmbH) were treated with buffered hydrofluoric acid and annealed at 950 °C for 1.5 hour in air. The terminations for various substrates and for films after growth were confirmed by atomic force microscopy.

### Film growth

Epitaxial LSTO films were grown on STO single-crystal substrates in a pulse laser deposition (PLD) system using a Lambda Physik KrF excimer laser with wavelength 248 nanometer, pulse width of approximately 15 ns and system base pressure of 10^−8^ Torr. The laser energy density was maintained at 1.6 J/cm^2^ with a frequency of 1 Hz for all the depositions. The layer-by-layer growth was monitored by *in situ* reflection high energy electron diffraction (RHEED) (see Fig. S1a for an example) with ~15 pulses per unit cell growth rate in this study. The temperature was set at 850 °C and the oxygen partial pressure was fixed at 10^−4^ Torr during the whole process including warming, deposition and cooling processes. Although the 10^−4^ Torr is not on the higher side of oxygen partial pressure, there is no obvious oxygen vacancies created during the growth. If there were oxygen vacancies, there could have been induced conductivity in all samples. However, there is no conductivity observed in thin LSTO/STO sample with LSTO thickness smaller than 6 uc. Similarly, there should be no conductivity from the diffusion of Sr or other elements as well. In addition, 10^−4^ Torr of oxygen partial pressure was also reported having no influence on the electrical conductivity^24^. After the growth, samples were cooled to room temperature in a ramping rate of 30 degree per minute. To demonstrate the atomic flat surface, an image measured by atomic force microscopy on 11 uc LSTO films on STO substrate is shown in Fig. S1b. The correct elemental composition and excellent crystalline quality were confirmed by Rutherford backscattering spectroscopy and channeling experiments (Fig. S3).

### Transport measurement

The sheet resistance *R*_S_ and the carrier concentration *n* were obtained as a function of temperature from 300 to 2 K by applying a d.c. current of 1 *μ*A. The measurement geometry was Van der Pauw geometry on square shaped samples (5 × 5 mm^2^). Ohmic contacts were formed by ultrasonically bonding Al wires. The method of ultrasonically bonding is a typical way of creating electrically contact to the interface 2DEG, due to its capability in penetrating through thick oxide layers, such as LaVO_3_^8^, Al_2_O_3_^10^ and LAO^12^,^17^,^43^. For the Hall measurements the magnetic field B was varied between ± 9 T.

### HLN fitting

WAL is characterized as a sharp suppression of resistance at low magnetic field. The expression of Eq. 4 is valid for 2D WAL in perpendicular magnetic fields^44^,^46^,^51^. The equation contains two free parameters, namely the prefactor α and the dephasing length *L*_φ_. The experimental data of MR used for fitting is the original MR data without background subtraction. The two parameters are obtained by fitting the experimental curves in MatLab. In the fitting, α were varied with a 0.5 interval without any range limitation and *L*_φ_ are kept completely free. In the process of fitting, the best fit of HLN is the fit with the minimum root mean square (RMS) value. In the Fig. 5c, the best fit and raw data are plotted together with fitted values of α^RMS^ and *L*_φ_^RMS^.

## Additional Information

**How to cite this article**: Renshaw Wang, X. *et al*. Parallel charge sheets of electron liquid and gas in La_0.5_Sr_0.5_TiO_3_/SrTiO_3_ heterostructures. *Sci. Rep*. **5**, 18282; doi: 10.1038/srep18282 (2015).

## Supplementary Material

Supplementary Information
