# Supplementary material for: Parallel charge sheets of electron liquid and gas in La0.5Sr0.5TiO3/SrTiO3 heterostructures
Source: Sci Rep. 2015 Dec 16;5:18282. doi: 10.1038/srep18282 (PMC4680910; doi:10.1038/srep18282)
Supplement: Supplementary Information [file srep18282-s1.pdf]

Supplementary Information

Parallel charge sheets of electron liquid and gas in $La_{0.5}Sr_{0.5}TiO_3/SrTiO_3$ heterostructures

X. Renshaw Wang[1,2,3,★,♯], L. Sun[1,★], Z. Huang[1,2], W. M. Lü[1,2], M. Motapothula[1,2], A. Annadi[1,2], Z. Q. Liu[1,2], S. W. Zeng[1,2], T. Venkatesan[1,2,4], and Ariando[1,2]

[1]*NUSNNI-Nanocore, National University of Singapore, 117411 Singapore.*

[2]*Department of Physics, National University of Singapore, 117542 Singapore.*

[3]*Faculty of Science and Technology and MESA+ Institute for Nanotechnology, University of Twente, P.O. BOX 217, 7500 AE Enschede, The Netherlands.*

[4]*Department of Electrical and Computer Engineering, National University of Singapore, 117576 Singapore.*

[♯]*Presently at Electrochemical Energy Laboratory, Massachusetts Institute of Technology, Cambridge, Massachusetts 02139, USA.*

[★]*These authors contributed equally to this work. Correspondence and requests for materials should be addressed to X. R. W. and A. (email: renshaw.wang@gmail.com & ariando@nus.edu.sg).*

I. Additional transport data

Figure S1c shows the sheet resistance of various thickness LSTO films on STO substrates. The insert shows a full range of sheet resistance for 6 uc LSTO film. Noted that the resistance of 6 uc LSTO film is larger than 25.8 kΩ, which is the quantum resistance for a two dimensional system. The quadratic sheet resistance (R_S) versus temperature curves indicates electron liquid (EL) behaviour. The slope of R_S versus T^2 curves reflects the electron-electron scattering intensity. This is shown in Fig. S1d after the thickness normalization.

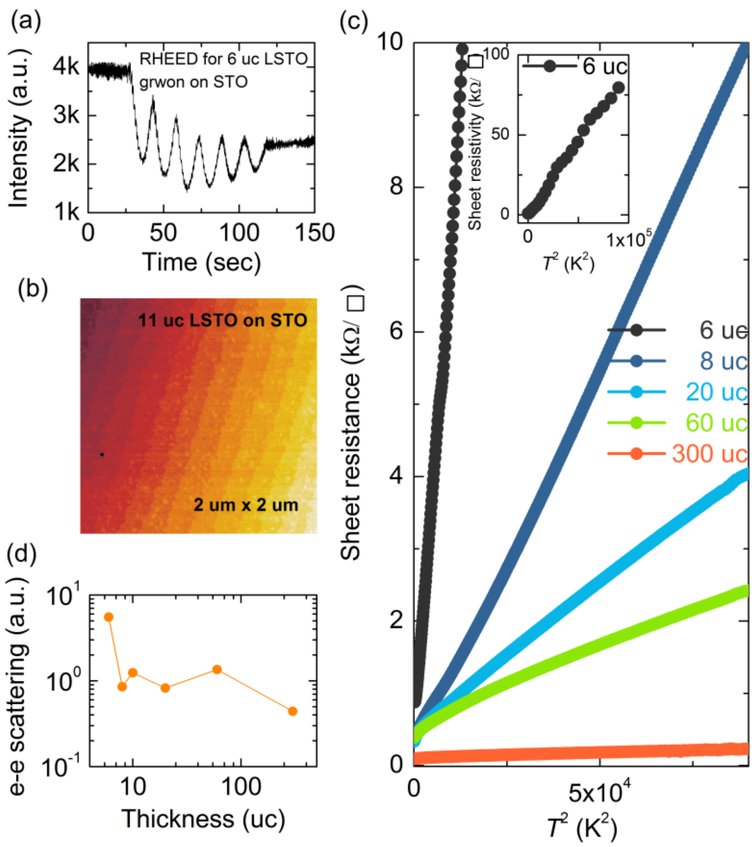

Figure S1. Sample preparation and basic characterizations. (a), RHEED oscillation example for an 11 uc LSTO grown on STO substrate. (b) Atomic force microscopy image for a typical 11 uc LSTO film. (c), The sheet resistance for LSTO with various thicknesses grown on STO substrate. (d) Electron-electron scattering intensity obtained from (a).

II. Linear Hall resistance (*R_{xy}*) in LSTO/LAO

Strikingly, among the LSTO grown on different substrates, only LSTO/STO heterosturctures exhibits nonlinear Hall effect. As shown in Fig. S2a and S2b, a strong nonlinear Hall effect as function of magnetic field was observed in LSTO/STO heterostructures at temperatures below 100 K, whereas it is always linear for the LSTO/LAO heterostructures from 300 to 10 K. This observation indicates that LSTO is a single band conducting material and only one type of carrier is expected. To confirm the location of the 2DEG, field effect studies were performed on 300 uc LSTO film, and a nonlinear Hall resistance was demonstrated. During the measurement, the

electric field was applied from the backside of the 0.5 mm thick STO substrate, the leakage current from drain to gate was maintained well below 100 nA range and the source-drain current was also fixed at 1 μA. In Fig. S2c, the transport properties under different back gate fields are shown. The nonlinear Hall resistance is found to be tunable by the back gate voltage and the *n* of 2DEG indeed increases when increasing the positive back gate voltage. Following the same analysis introduced in the main text, the 2D channel is thus confirmed to be at the interface between STO and LSTO (see Fig. S2d).

Moreover, the low carrier density and high mobility carriers has mobility ~ 2000. Since the mobility is higher than the weak field limit discussed in the main text, this type of high mobility carriers may exhibit quantum effect at lower temperature where thermal effect is negligible.

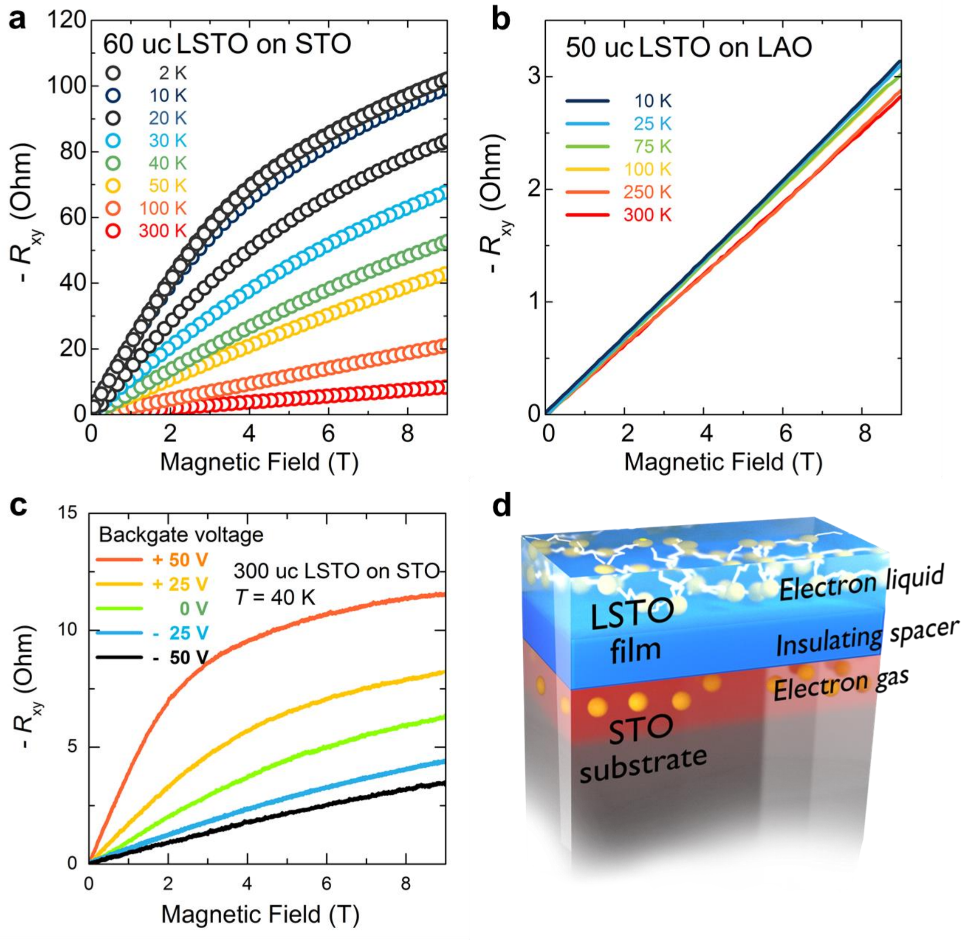

Figure S2. Coexistence of two types of carriers in LSTO/STO heterostructures. R_{xy} for LSTO/STO heterostructures (a) and LSTO/LAO heterostructures (b) at

different temperatures. Nonlinear R_{xy} is only observed in LSTO/STO heterostructure at a temperature below ~100 K. (c) Back gate experiment on 300 uc LSTO/STO heterostructures. A suggested coexistence of two types of carriers is schematically shown in (d). In the schematic drawing, red region represents electron gas and blue region represents LSTO material. Yellow balls represents electrons and the white lines between electrons represents electron-electron interactions.

III. Rutherford Backscattering-Channeling Spectroscopy

Because La percentage is as high as 50%, a detailed careful investigation on stoichiometry, crystalline structure and La substitution is highly important. In Fig. S3, a typical Rutherford backscattering (RBS)/Ion channeling spectrum on 200 uc LSTO film on STO is demonstrated. The 2.7 % channeling minimum yield for La in LSTO film demonstrates the well crystal quality of the films and the good La substitution of Sr. The expected La/Sr ratio was quantitatively confirmed by a simulated curve of LSTO (x=0.5).

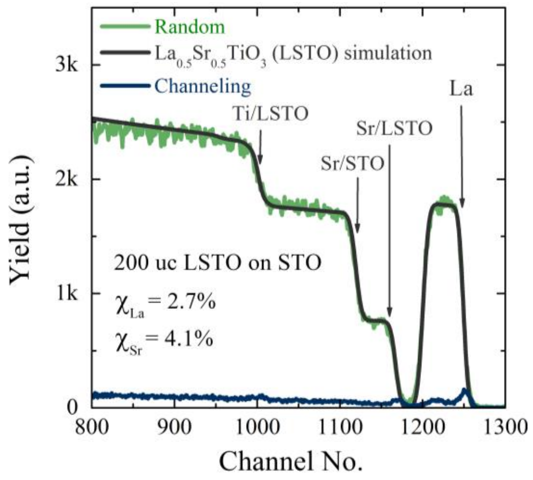

Figure S3. RBS Channeling on 200 uc LSTO film. The channeling minimum yield for La and Sr are 2.7% and 4.7% respectively indicates good La substitution. The measured random matches well with simulated LSTO curve proving the correct composition.

IV. Discussion on magnetism in LSTO films

The nonlinear HR does not originate from ferromagnetism in LSTO based on the facts that 1) no hysteresis loop is observed in HR and MR data and 2) no stray field is detected by scanning SQUID microscopy.

Figure S4 shows a zoom-in of HR of 60 uc LSTO grown on STO at low field and measured at 2 K. Within our detection limit, there is no hysteresis loop is observed in HR data. The MR data presented in main text Fig. 5 also show no hysteresis, indicating no ferromagnetism in LSTO. In addition, even assuming there is ferromagnetism which cannot be detected by our transport measurements, it will be too weak to product noticeable effect on the nonlinear HR.

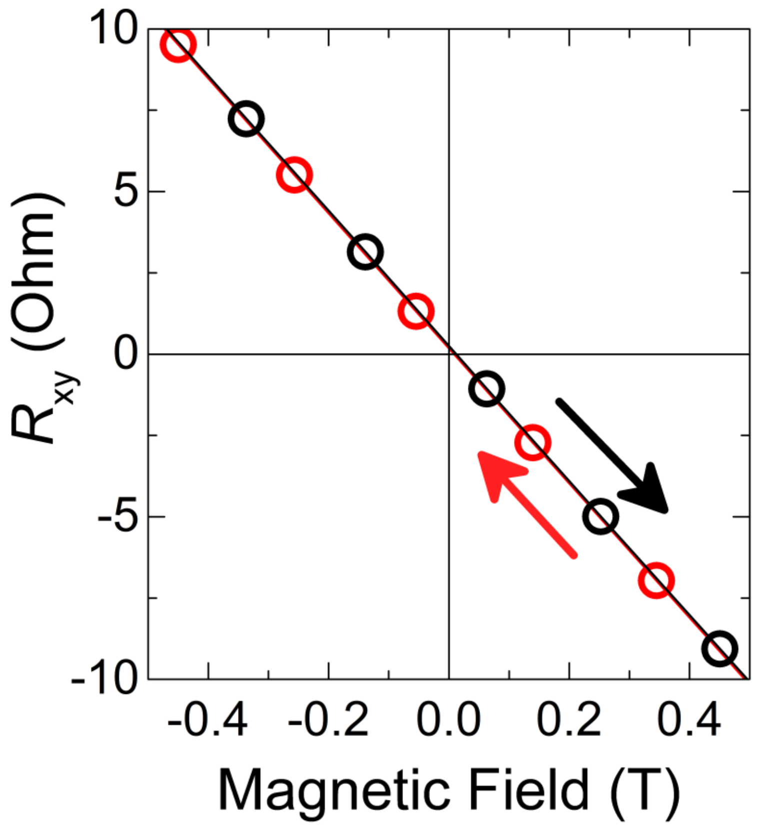

Figure S4. No hysteresis loop observed in 60 uc LSTO grown on STO at 2 K.

Magnetism of LSTO is also measured by scanning SQUID microscopy at 4 K. Figure S5 shows a 70 um by 200 um scan on 200 uc LSTO film grown on STO with a spatical resolution of 1 um by 1 um. Magnetic filed intensity was measured by measuring magnetic flux passing through a SQUID pickup loop. Warm color in figure represents

the intensity magnetic filed passing through pickup loop in one direction and cold color in figure represents the intensity of magnetic field passing through pickup up in the other direction. As shown in Fig. S5, magnetic field intensity variation is within noise level of the scan, demonstrating no stray field originating from ferromagnetic domain. Since scanning SQUID detects magnetic flux which can be generated from both in-plane and out-of-plane magnetic domain, the 200 uc LSTO is not ferromagnetic.

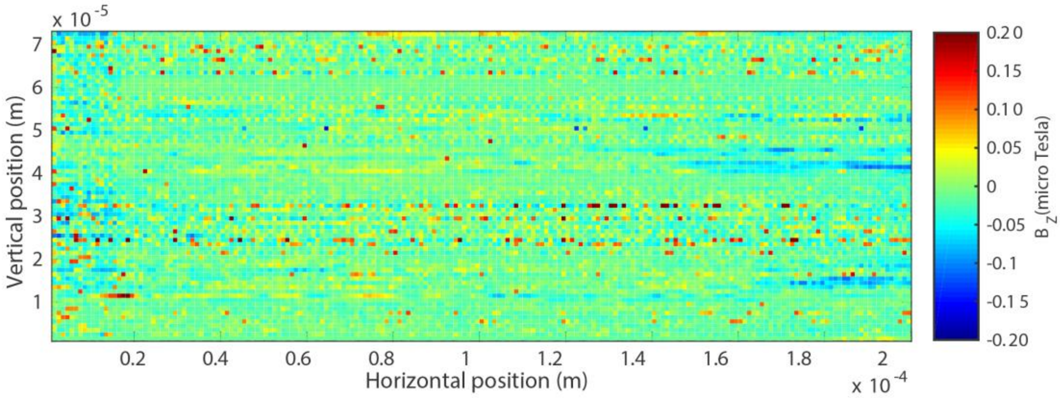

Figure S5. Non-ferromagnetic 200 uc LSTO imaged by scanning SQUID microscopy at 4 K. The image is a 70 um by 200 um image of magnetic field intensity distribution with a pixel resolution of 1 um by 1 um. Magnetic field intensity is within noise level of the measurement.

V. Representative HLN fits

The HLN fit was conducted in MatLab by traversing both α and L_{φ}^{RMS}. The best fit is determined by the minimum RMS value which is the root mean square between fitted curve and measured data. All possible combinations of α^{RMS} and L_{φ}^{RMS} are calculated. Consequently, α^{RMS} = -1 and L_{φ}^{RMS} = 136 nm give the global minimum, indicating the best fit possible. In order to present accuracy of the HLN fit, fig. S6 shows three most relevant fits.

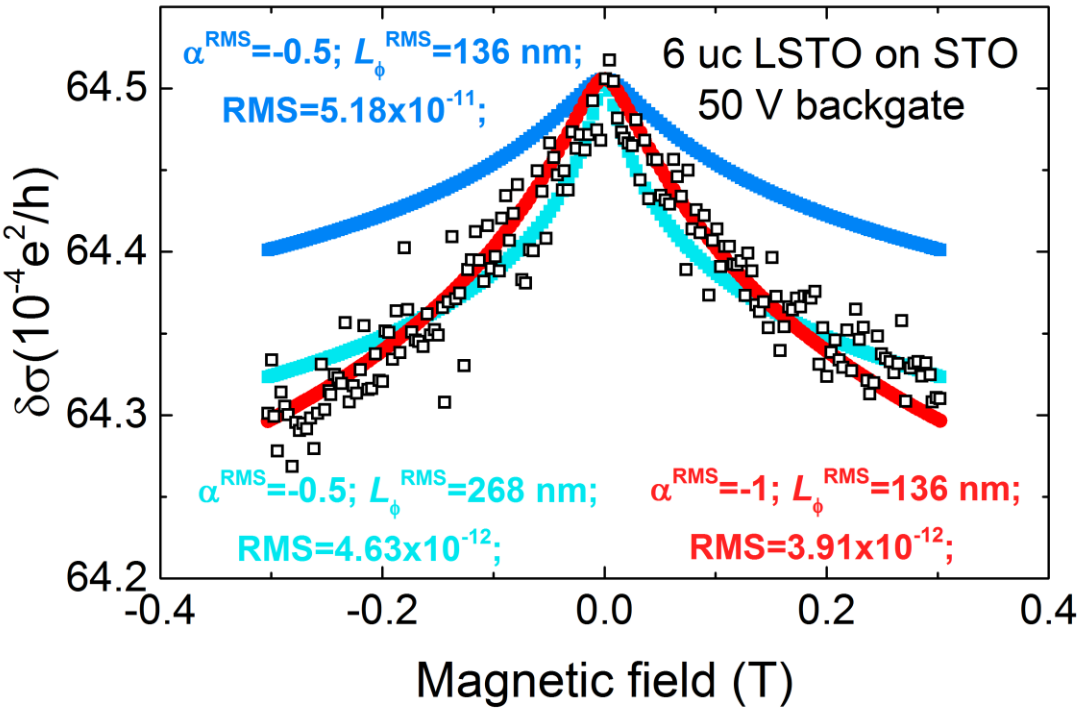

Figure S6. Representative fits of HLN fits with different parameters on 6 uc LSTO grown on STO under 50 V back gate voltage. The RMS is the room mean square value of fitted value versus measured data. Considering RMS value, □RMS = -1 and LφRMS = 136 nm is the best fits for 6 uc LSTO on STO under 50 V backgate voltage.
